# Evaluation of Cement Remaining After Debonding and Polishing in Lingual Multibracket Appliance Using Planning Imaging 3D Software

**DOI:** 10.3390/ma18040781

**Published:** 2025-02-11

**Authors:** Alba Belanche Monterde, Javier Flores-Fraile, Jorge Alonso Pérez-Barquero, Andrea Peiro-Aubalat, Patricia Mendieta Lasierra, Álvaro Zubizarreta-Macho

**Affiliations:** 1 Faculty of Medicine, University of Salamanca, 37008 Salamanca, Spain; belanche.alba@usal.es (A.B.M.); alvarozubizarreta@usal.es (Á.Z.-M.); 2 Department of Stomatology, Faculty of Medicine and Dentistry, University of Valencia, 46010 Valencia, Spain; jorgealonso86@gmail.com; 3 Department of Biomedicine, Faculty of Medicine and Health Sciences, University of Barcelona, 08007 Barcelona, Spain; apeiroau68@alumnes.ub.es; 4 Department of Medicine and Medical Specialties, Faculty of Health Sciences, University of Alcalá de Henares, 28801 Madrid, Spain; patricia.mendietal@edu.uah.es; 5 Department of Implant Surgery, Faculty of Health Sciences, Alfonso X El Sabio University, 28691 Madrid, Spain

**Keywords:** orthodontics, remaining cement, lingual multibracket appliance, intraoral scanner

## Abstract

Background: The aim of the present study was to assess the accuracy, repeatability and reproducibility of a novel digital technique to analyze the remaining cement after debonding lingual multibracket appliances and after polishing the remaining cement. Methods: Thirteen teeth were embedded in an epoxy resin simulating a dental arch and subsequently a digital impression was taken using an intraoral scanner; obtaining a standard tessellation language (STL1) digital file. Lingual multibracket appliances were bonded and debonded on the lingual surfaces of all teeth and another digital impression was made (STL2). Finally, the polishing procedure of the remaining cement was performed and a digital impression was taken (STL3). The teeth were individually segmented from the digital files and an alignment was performed between STL1 and STL2 and between STL1 and STL3 digital files using specific cephalometric 2D/3D software to assess the remaining cement after debonding and after polishing lingual multibracket appliances. The reproducibility and repeatability capacity of the measurement digital method was assessed using a Gage R&R statistical analysis. Results: The results were assessed by a statistical program and showed a statistically significant (*p* < 0.001) decrease in weight, thickness, and height of the cement remaining after polishing, with a mean decrease of 2.09 mm in width (X plane), 0.12 mm in thickness (Y plane), and 1.87 mm in height (Z plane). Conclusions: The use of novel digital diagnosis software is a repeatable, reproducible, and accurate measurement technique to analyze the remaining cement after debonding lingual multibracket appliances and after polishing the remaining cement. Practical Implications: The diagnosis cephalometric software evaluation of the polishing technique with different materials, burs or polishing sequences brings the research closer to the clinical field. This methodology allows the orthodontist to clinically measure the cement remnants after polishing without the need for teeth extraction and with current clinic objects such as intraoral scanning and orthodontic cephalometric software. This might give orthodontics more clearance in terms of better burs or protocols for polishing.

## 1. Introduction

Nowadays, the aesthetic requirements of society are greater and more aesthetic orthodontic options have emerged, such as use of aligners and lingual multibracket appliances [1]. In addition, lingual multibracket appliances are recommended to prevent the formation of white spot lesions, which are associated with vestibular multibracket appliances [2]. The micro- and macroanatomical differences of the lingual surfaces of teeth with respect to the vestibular surfaces must be taken into account. The lingual surfaces are less rough than the buccal surfaces and acid etching produces less dissolution of enamel prisms on the lingual than on buccal surfaces [3] and the macroanatomy, in particular of the incisors, is more irregular. In addition, the field of vision and the access during lingual multibracket therapy is worse, which may make difficult adhesion and/or the use of polishing techniques [4]. Despite these anatomical differences, there has not been a notable increase in statistical failures in bonding between lingual compared to vestibular multibracket appliances, although the rate of bonding failure in lingual brackets seems to increase when acid etching is not performed, despite use of self-etching adhesive systems [5,6]. In addition, it has been observed that self-ligating brackets lead to better quality oral health after bonding that conventional brackets, so the type of bracket might affect to the periodontal status [7]. It has also been noted that fluoride-based cements have higher sear bond strength (SBS) than conventional cements in lingual multibracket appliances, so these have been recommended [8]. It has been observed that resin-modified glass ionomer cements do not allow better bonding outcomes than current composites for brackets. Adhesion problems have been solved by indirect bonding in lingual orthodontics [9]. Moreover, in lingual orthodontics the fact that this technique has biomechanical differences seems important. Lingual multibrackets systems have less distance between the point of application of the forces due to their lower interbracket distances in contrast to vestibular orthodontics. That might allow more effective application but also to difficulties in finishing, which is the reason why lingual orthodontics is normally customized and individualized [10]. In a previous study, it was found that a lower volume of cement remained after debonding and after polishing in lingual multibracket appliances than in vestibular multibracket appliances, measured with the geomorphometry technique [11]. In contrast, Sfodrini et al. obtained lower values in the adhesive remnant index (ARI) in vestibular multibracket appliances than in lingual multibracket appliances [12]. Kuskonmaz et al. observed with laser confocal scanning microscopy that 41% of the area of the mesh was covered by cement after debonding lingual brackets and more area of cement remaining was observed on the mesh of lingual molar brackets [13]. In the present study, the cephalometric software Dolphin version 11.9 (1 June 2022) was used to assess the cement remaining after debonding and after the use of polishing techniques in lingual multibracket appliances, taking into account that cephalometric software analysis is vital in orthodontics to perform a correct diagnosis, allowing evaluation of the height and position of maxillaries, teeth, and associated craniofacial structures, and anticipated changes during growth [14]. Cement remnants after brackets debonding and polishing have been studied with different methodologies such us scanning electron microscopy, profilometry, atomic force microscopy, and optical coherence tomography technology. These technologies allow measurement of the cement remnants but the teeth must be extracted to be introduced into the machine. Another methodology more widely used to evaluate cement remnants is the adhesive remnant index (ARI), while other indexes focus on the subjective evaluation of the remnants by the operator [15].

The aim of the present study was to assess maximum measurements of cement remaining after debonding lingual multibracket appliances and cement remaining after polishing the lingual surfaces evaluated in the three planes of space.

## 2. Methods

### 2.1. Study Design

The present study was performed using 13 extracted upper maxillaries teeth from all dental sectors. We selected teeth that were unfractured, without caries, restorations or prosthesis, even though they were not extracted because of conservative or endodontic reasons. Teeth with restoration or caries were excluded for this trial. Only orthodontically or periodontally extracted teeth were included in the trial. The sample was selected at Alfonso X El Sabio University (Madrid, Spain), Alfonso X El Sabio University (Madrid, Spain), and the Clinical Master Degree in Orthodontics at University of Salamanca (Salamanca, Spain) between September and November 2020. A randomized controlled trial was conducted based on the German Ethics Committee’s statement for the use of organic tissues in medical research [16]. And was authorized by the Ethical Committee of the Faculty of Health Sciences, University Alfonso X el Sabio (Madrid, Spain) Faculty of Health Sciences, University Alfonso X el Sabio (Madrid, Spain) in July 2020 (Process No.08/2020). After giving informed consent, the patients agreed to allow use of their teeth.

### 2.2. Experimental Procedure

For the study, an epoxy resin model (Ref.: 20-8130-128. EpoxiCure^®^, Buehler, Lake Bluff, IL, USA) was made and the selected teeth were introduced and located in the corresponding position to simulate a superior dental arch. Next, an initial scanner (STL1) with an intraoral scanner (True Definition, 3M ESPE™, Saint Paul, MN, USA) was used to produce digital images in standard tessellation language file.

Fixed lingual multibracket appliances were bonded in the lingual enamel surfaces from 15 to 25 teeth and tubes were bonded in molars (Discovery^®^ Delight, Dentaurum GmbH & Co. KG., Ispringen, Germany) by a unique operator with a three-step adhesive technique, after previous etching with 37% orthophosphoric acid (Ortho Solo™, Ormco Corporation, Brea, CA, USA) for 20 s, which was removed using a washing and drying procedure. Then, a photo-polymerized resin primer was carefully applied (Unitek Transbond™ XT, 3M ESPE™, Saint Paul, MN, USA) with a microbrush (Plus slim, Microbrush International, Grafton, MA, USA) and it was finally photopolymerized (BluePhase G2™, Ivoclar Vivadent, Schaan, Principado de Liechtenstein) for 20 s. The bonding resin agent was used following the manufacturer recommendations (Transbond™ XT, 3M ESPE™, Saint Paul, MN, USA). Then, the lingual brackets were debonded using a debonding Weingart plier (Carl Martin, Solingen, Germany), and afterwards polishing was performed on the lingual surfaces using a micro-motor handpiece (INTRA MULTIflex L181M, KaVo, Biberach an der Riss, Germany) and a polishing composite bur (H379AGK, KOMET Dental, Lemgo, Germany) with water irrigation until there were no more visible remnants on the surfaces to the clinician’s eye. Finally, another digital impression (True Definition, 3M ESPE™, Saint Paul, MN, USA) was taken to evaluate the effectiveness of the polishing (STL3). The operator performed the polishing by naked eye, taking into account the adhesive remnant index (ARI) and decided on one or two stages of ARI polishing for each tooth. A second operator confirmed the polishing. Both operator were experienced orthodontists.

### 2.3. Alignment Procedure

**Figure 1:**
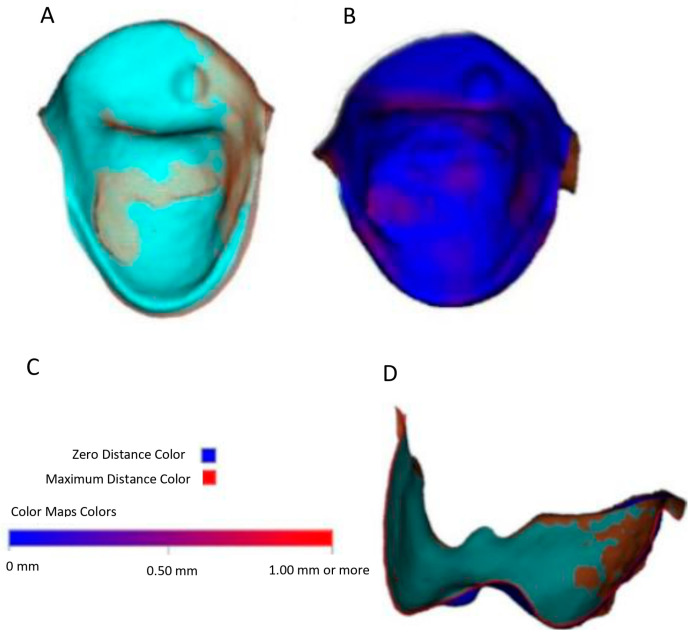
Overlay of STL1 with STL2. Frontal view of cement remaining on the lingual surface of 1.5 teeth (**A**). Frontal view of lingual surface of 1.5 with ColorMap tool (**B**). Precision scale and legend of the ColorMap tool used (**C**). Sagittal view of the overlay in 15 teeth with outlines of STL in red (**D**).

STL digital files 1 to 4 were uploaded into the Dolphin Imaging 3D cephalometric software in a new clinical record created for the study with specific folders created corresponding to each specific STL. The teeth of STL1 to STL4 were segmented using the sculpting tool available in Dolphin Imaging 3D with the aim of avoiding the errors that may be produced during posterior alignment. When segmented digital files of each tooth were obtained, they were exported into computer storage, obtaining STL digital files specific to each tooth and each moment of cementation. Next, the STL1 with STL 3 digital files were firstly aligned and measured and after that, STL 1 and STL 4 digital files were also aligned and measured to analyze linear differences. An initial superposition by dots was performed by placing 3 dots at the same location of the tridimensional space in both STL digital files. Then, we obtained a more accurate superposition using an application available in Dolphin Imaging 3D software named ColorMap (version 11.9), which detects minimal distances, overlaying changes to red color when distances are bigger than 1 mm and in blue color when distances are close to 0 between the digital images (Figure 1C). Overlays were made with ColorMap until the buccal surface was totally blue; however, in the lingual surfaces red zones were observed due to the cement remains (Figure 1B). In order to ensure a correct superposition of the STLs, the superpositions after use of ColorMap were visualized in sagittal and coronal slices evaluating the distances between the undisturbed vestibular surfaces, making small modifications when necessary, since the distances to be handled in this study were relatively small (Figure 1D).

### 2.4. Measurement Procedure

**Figure 2:**
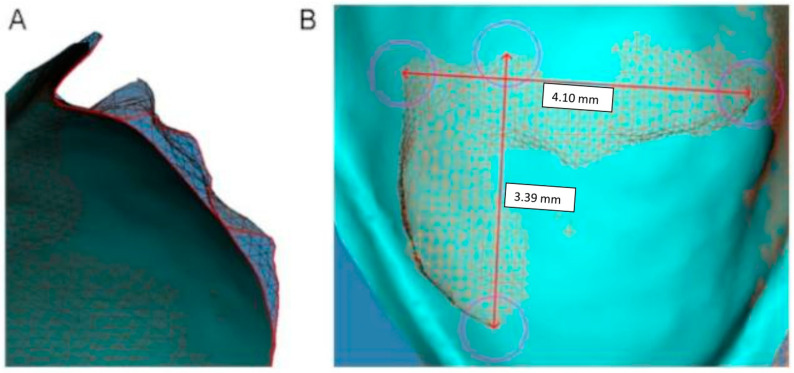
Measures obtained in STL1 with STL 2 overlay. Sagittal view of superimposition with STL2 observed in tessellation image (**A**). Measures of height and width of the cement remaining in a frontal view of lingual surface of 1.5 teeth after debonding (**B**).

Once the STLs were assessed to have the best overlay possible, the images superimposed (STL2, STL3) were changed into tessellation observation in order to obtain a more accurate measurement in the 3D image. The lineal measures of cement to evaluate were obtained by use of the 2D line tool in Dolphin Imaging 3D software, measuring the greatest distance in height and width in a frontal vision of lingual surfaces (Figure 2B). The thickness of the volume of cement remaining was also measured from a sagittal view of the overlays STL1–2 and STL1–3 of each tooth, taking the greatest thickness obtained for the analysis.

The results obtained of cement remaining after debonding of lingual multibracket appliances and the cement remaining after polishing were expressed on three planes of space (X,Y,Z), X being the maximum width, Y the maximum thickness, and Z the maximum height of the cement remains after debonding, and X′,Y′,Z′ the corresponding measurements of cement remaining after the polishing technique.

### 2.5. Validation of Repeatability and Reproductibility

To validate the repeatability and reproducibility of this method of measuring cement remaining after debonding and after polishing using the Dolphin 3D cephalometric imaging software, we randomly selected 10 teeth and measurements were taken twice by two operators (Operator A and Operator B). Then, a Gage R&R statistical analysis was performed to validate the repeatability and reproducibility of the technique. This technique allows the clinician to evaluate polishing techniques in real situations due to easy application. In addition, 3D Dolphin cephalometric software is a current software widely used by orthodontists, in contrast to other methodologies. In addition, the current methodologies that measure cement remnants require extracted teeth, while this methodology based on STL measurement does not.

### 2.6. Statistical Tests

The variables were created in the statistical software (SPSS 22.00, Microsoft Inc., Redmond, WA, USA) and the measures obtained corresponding to the variables were introduced. A descriptive and comparative statistical analysis was made to compare the means of the variables and the standard deviation (SD) obtained. We compared the means of STL 1–3 overlays and STL 1–4 overlays in order to evaluate the cement remains on the lingual surfaces, and also performed statistical analysis to compare anterior to posterior polishing between STL1–3 and STL1–4. The statistical significance was set at *p* < 0.05.

## 3. Results

The mean, minimum, maximum, and standard deviation (SD) values corresponding to maximum distances in width (X), thickness (Y), and height (Z) of cement remaining after debonding (STL1–3) are presented on Table 1.

The mean, minimum, maximum, and standard deviation (SD) values corresponding to maximum distances in width (X′), thickness (Y′), and height (Z′) of cement remaining after polishing (STL1–4) are presented on Table 2.

For comparative analysis, the differences between the measurements obtained after debonding and after polishing were calculated for width (X-X′), thickness (Y-Y′), and heigh (Z-Z′) individually. Afterwards, we analyzed the condition of the applications against the different comparative tests with a normality test (Shapiro-Wilk normality test). The appropriate linear or non-parametric model was applied depending on the fulfillment of the application criteria (*t*-test for paired data or Wilcoxon’s rank test). Statistically significant differences were observed between the remaining cement after debonding fixed lingual multibracket appliances in STL1 and STL3 digital files in the X-plane (*p* ≤ 0.001), Y-plane (*p* ≤ 0.001), and Z-plane (*p* ≤ 0.001).

Statistically significant differences were observed in the cement remaining after debonding and after polishing in fixed lingual multibracket appliances in the X-plane (*p* = 0.001), Y-plane (*p* <0.001), and Z-plane (*p* < 0.001).

In the anterior sector of the arch, statistically significant differences were observed in the difference of the cement remaining after debonding and after the polishing technique in the X-plane (*p* = 0.04) and Y-plane (*p* < 0.001) but no statistically significant differences were observed in the Z-plane (*p* = 0.051). In the posterior sector of the arch, statistically significant differences were observed in the cement remaining after debonding and after the polishing technique in fixed lingual multibracket appliances in the X-plane (*p* = 0.02), Y-plane (*p* = 0.016), and in the Z-plane (*p* = 0.016) (Table 3 and Table 4).

**Figure 3:**
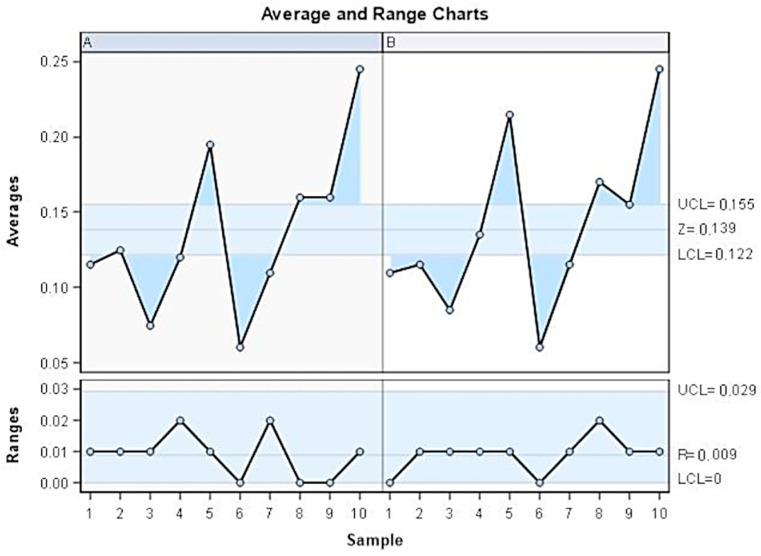
Descriptive statistics of the difference between cement remaining after debonding and after the polishing technique in each plane of space (mm). Color blue shows similar measurements between operators. (**A**) First operator measurements. (**B**) Second operator measurements.

**Figure 4:**
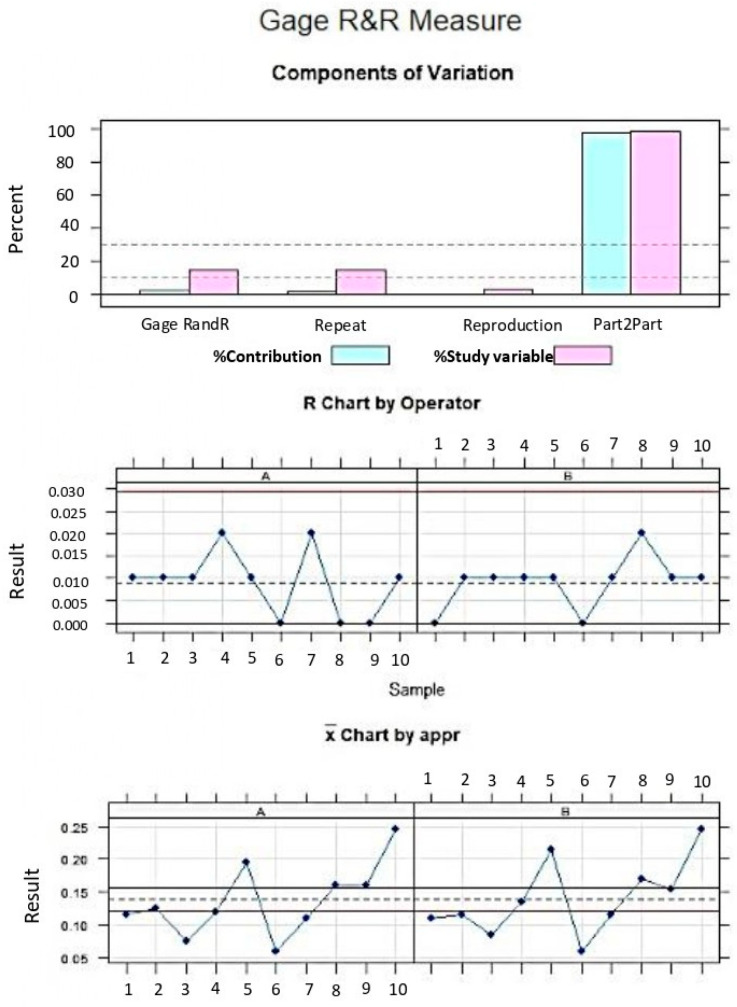
Evaluation chart of cement measurements indicating the difference of the two measurements of each observer to assess the contribution of each variable to the total of variation obtained (component of variation) with a mean control chart (R Chart by Operator), measurement points in the graph (result by operator), and the interactions (operation interactions). The values are within the confidence limits. A and B represents both operator measurements.

The GageR&R analysis of the method of measurement of cement remaining with the software used was based on the measures of cement remaining and showed a repeatability percentage of 2.2% and a reproducibility percentage of 0.1%. The values obtained showed repeatability (<10%) and high reproducibility (<1%) of the method used (Figure 3 and Figure 4).

## 4. Discussion

In multibracket appliances, it is relative common to study cement remaining using indexes such as the adhesive remnant index (ARI) based on the direct visualization of the cement remaining and assigning a score considering subjectively the quantity of cement remaining. This was demonstrated not to be reproducible between operators [10,13]. Some authors use laser confocal scanning to have more accuracy in the assessment of cement remaining by this and other observational indexes [11]. Morphometry method has been recently presented as an accurate, reproducible, and repeatable method of obtaining volumes and areas of cement remaining in buccal and in lingual multibracket appliances [17,18]. The area of cement remaining on surfaces has a direct relation with the SBS, and showed that SBS decreased when the area of cement remaining was higher and the enamel loss was higher when the mesh was free of cement remnants [19]. In a previous study performed with morphometry, it was observed that there were significantly smaller volumes of cement remaining in lingual multibracket appliances than in vestibular multibracket appliances after debonding (*p* = 0.002) and after polishing techniques (*p* = 0.004). The mean volume obtained in lingual multibracket appliances was 0.87 ± 1.34 mm^3^ after debonding and 0.13 ± 0.15 mm^3^ after polishing, and with vestibular multibracket appliances the mean values obtained were 3.48 ± 0.96 mm^3^ and 0.70 ± 0.56 mm^3^, respectively [9]. In the present study, it was noticed that polishing the surfaces after debonding in lingual multibracket appliance was effective since statistically significant decreases were found between the initial and final cement remaining measured in the three planes of space corresponding with width (X-plane), thickness (Y-plane), and height (Z-plane). The cement remaining decreased after polishing 2.1 mm in width, 0.10 mm in thickness, and 1.9 mm in height in median values, however the decrease in width and height were similar, while in thickness the difference was smaller because the measurements found in thickness after debonding were much lower. Moreover, when the cement remaining was assessed by segmenting into the anterior or posterior sector, statistically significant differences (*p* < 0.05) were found in the three planes of the space in the posterior sector. However, in the anterior sector there were no statistically significant differences in height between the cement after debonding and after polishing (*p* > 0.05). After the polishing technique, cement remains were sometimes found as a consequence of the chromatic similarity of the bonding agents used to perform bracket adhesion, so florescence-aided identification technique (FIT) was used to obtain a better visualization of cement remaining based on the resin fluorescence proprieties of the resins used in cements [20]. However, the studies showed that changes in color (ΔE) were perceivable by spectrophotometer after debonding and they were associated with the length of the resin tags conformed during bracket adhesion [21]. Hence, the removal of the cement remaining is necessary but it produces different degrees of enamel depending on the material used for polishing being greater when tungsten burs are used [22]. Enamel roughness is increased after debonding so the polishing should be able to reduce that roughness and restore it most similarly to pretreatment values. Enamel roughness increase might lead to decolorization and plaque retention after treatment so this should be taken into account because of carious incidences. Another problems associated with orthodontics are white spot lesions that normally have higher prevalence in vestibular orthodontics due to the tongue cleaning aspect in lingual surfaces [23,24].

On the other hand, (3D) imaging planning software is well-established and accepted in areas of dentistry such us surgery and implantology, endodontics or orthodontics, and these 3D imaging planning software packages allow obtaining accurate lineal measures, two-dimensional (2D) areas, and 3D volumes. Nowadays, new automated methods for multimodal registration of intraoral scanning have been developed with a time reduction of the registration. These methods provide accurate images and integration without operator biases in orthodontics [25]. The advances in digital dentistry have increased the efficiency and accuracy of 3D imaging and artificial intelligence has also shown interesting results in registrations [26]. In orthodontics, manual 2D measures were first used to perform cephalometry procedures, but this method was long and commonly entailed magnification or measuring errors; in recent decades, with the emergence and improvement of informatics, cephalometry has become computer assisted [27]. These 2D imaging planning software packages allow obtaining angles or linear measures and making comparisons by superposition procedures; however, controversial results were obtained comparing the accuracy of digital and manual cephalometric procedures due to calibration, pixel size, and low image quality resolution [28]. In addition, the development and improvement of digital radiographic techniques have increased the reproducibility of cephalometric procedures using less radiation exposure and providing higher accuracy rates comparing to conventional radiographies. Moreover, Polat-Ozsoy et al. reported statistically significant differences in the reproducibility of digital cephalometric procedures in comparison with manual cephalometric procedures; however, the differences were clinically irrelevant. Nowadays, digital cephalometric software allows the alignment and subsequent analysis between digital files obtained by cone-beam computed tomography (CBCT) scan and 3D images obtained from intraoral scans [29]. In addition, these digital cephalometric software packages have also been used to measure the volume of nasal cavity, sinus, oropharynx, and nasopharynx [30]. Furthermore, other measurement procedures have been used to measure lineal distances of remaining cement; for example, the scanning electron microscope (SEM) and the optical coherence tomography (OCT) [15]. OCT images are able to be evaluated by automated method analysis. This methodology allows assessing the quality of polishing by measuring the enamel thickness by fully-automated image reconstruction of image sequences. This methodology requires even less time to assess polishing and 5 s only are needed for measuring teeth enamel thickness in quantity [31].

In addition, differences between posterior and anterior polishing in lingual orthodontics were found in this trial. These findings were attributed to the anatomical differences in convexity incisors and canines in contrast to the vestibular surface. The lingual surface of incisors shows the most inter- and intraindividual variability and highest differences are found in the cingle [32]. Also, lingual orthodontics are commonly bonded more apically than vestibular brackets due to the biomechanics that results in normal bonding to the lingual cingle [33].

The limitations of this trial are that it was an in vitro study and the methodology of measurement should be proven in real situations in which oral saliva and the difficulties of access to the lingual surfaces are real. However, as it was a measurement of intraoral scanning images, it might be equally applied to real environments. Further studies in vivo and with longer sample sizes will be needed for higher quality conclusions. In addition, it should be taken into account that the polishing technique has an operator risk of bias due to the high manual variation that affects polishing of lingual surfaces.

## 5. Conclusions

In conclusion, within the limitations of the study, polishing reduces significantly the cement remaining in the three planes of space in lingual multibracket appliances, however the reduction in height of anterior teeth may be insufficient. This methodology allows the clinician to perform an evaluation of cement remnants after bracket debonding.

## Figures and Tables

**Table 1:** Descriptive statistics of the maximum width, thickness, and height of cement remaining after debonding (mm).

	*n*	Mean	SD	Minimum	Maximum
X	13	2.70 ^a^	1.62	0.55	5.92
Y	13	0.18 ^b^	0.16	0.08	0.37
Z	13	2.90 ^c^	2.00	0.01	5.92

SD: standard deviation. ^a,b,c^ Statistically significant differences between groups (*p* < 0.05).

**Table 2:** Descriptive statistics of the maximum width, thickness, and height of cement remaining after the polishing technique (mm).

	*n*	Mean	SD	Minimum	Maximum
X′	13	0.61 ^a^	0.56	0.00	1.60
Y′	13	0.06 ^b^	0.06	0.00	0.16
Z′	13	1.03 ^c^	1.31	0.00	4.13

SD: standard deviation. ^a,b,c^ Statistically significant differences between groups (*p* < 0.05).

**Table 3:** Descriptive statistics of the difference between cement remaining after debonding and after the polishing technique in each plane of space (mm).

	*n*	Mean	Median	SD	Minimum	Maximum	*p*-Value
X-X′	13	2.09 ^a^	2.00	1.74	0.01	5.92	=0.001
Y-Y′	13	0.12 ^b^	0.10	0.12	0.02	0.33	<0.001
Z-Z′	13	1.87 ^c^	1.71	1.31	0.4	5.14	<0.001

SD: standard deviation. ^a,b,c^ Statistically significant differences between groups (*p* < 0.05).

**Table 4:** Descriptive statistics of the difference in cement remaining in each plane of space between debonding and after the polishing technique segmented by anterior or posterior sector of the arch (mm).

	*n*	Sector	Mean	SD	Minimum	Maximum	*p*-Value
X-X′	6	Anterior	2.24	1.99	0.76	5.92	=0.04
	7	Posterior	1.97	1.66	0.01	4.28	=0.02
Y-Y′	6	Anterior	0.10	0.02	0.08	0.14	<0.001 ^a^
	7	Posterior	0.13	0.14	0.02	0.33	=0.016
Z-Z′	6	Anterior	1.95	1.88	0.40	5.14	=0.051
	7	Posterior	1.81	0.66	0.82	2.92	=0.016

SD: standard deviation. ^a^ Statistically significant differences between groups (*p* < 0.05).

## Data Availability

The original contributions presented in this study are included in the article. Further inquiries can be directed to the corresponding author.
